# Physical activity‐based interventions in the management of dementia or cognitive impairment in sub‐Saharan Africa

**DOI:** 10.1002/alz70858_102579

**Published:** 2025-12-25

**Authors:** Michael C Ibekaku, Lori Weeks, Parisa Ghanouni, Lawrence A Adebusoye, Nazanin Nasiri, Chukwuebuka P Onyekere, Caitlin McArthur

**Affiliations:** ^1^ Dalhousie University, Halifax, NS, Canada; ^2^ Chief Tony Anenih Geriatric Centre, University College Hospital, Ibadan, Ibadan, Oyo State, Nigeria; ^3^ York University, York, ON, Canada

## Abstract

**Background:**

The number of people living with dementia globally is rising significantly. The World Alzheimer's report estimated that about 5.5% of the population older adults in sub‐Saharan Africa (SSA) are currently living with dementia, this is projected to rise significantly to 250% by 2050. There is increasing evidence that physical activity‐based interventions improve health outcomes in individuals with dementia. Recently, studies in SSA countries have been exploring the use of physical activity in dementia with varying outcomes, hence, a review of how physical activity has been utilized in dementia care is timely to summarize existing evidence and identify key characteristic and factors associated with physical activity interventions in dementia care in SSA. This scoping review aims to map the existing literature on physical activity‐based interventions for dementia care in sub‐Saharan Africa.

**Method:**

The review will follow the JBI methodology for scoping reviews and adhere to the Preferred Reporting Items for Systematic reviews and Meta‐Analyses extension for Scoping Reviews guidelines. A comprehensive search for relevant literature was conducted in PubMed, CINAHL, PsycINFO, SPORTDiscus, Scopus, Embase, and African Journals Online databases. Grey literature sources, including Google Scholar and Database of African Theses and Dissertations—Research (DATAD‐R) were also searched. We will conduct a hand search of the reference lists of all articles that will be included in the full‐text screening. Studies conducted in SSA countries that focused on using physical activity interventions to improve health outcomes for people with dementia or cognitive impairment will be considered eligible for inclusion in the review. There will be no restrictions on the language and date of publication.

**Result:**

A total of 2852 articles were retrieved from the databases searches with 2515 included in ongoing title and abstract after removal of duplicates. After hand search and full‐text screening, Data will be charted and summarized descriptively, with the results presented in narrative and visual forms.

**Conclusion:**

The findings from this scoping review will highlight the current state of physical activity‐ interventions for dementia management in sub‐Saharan Africa and highlight research priority areas.

